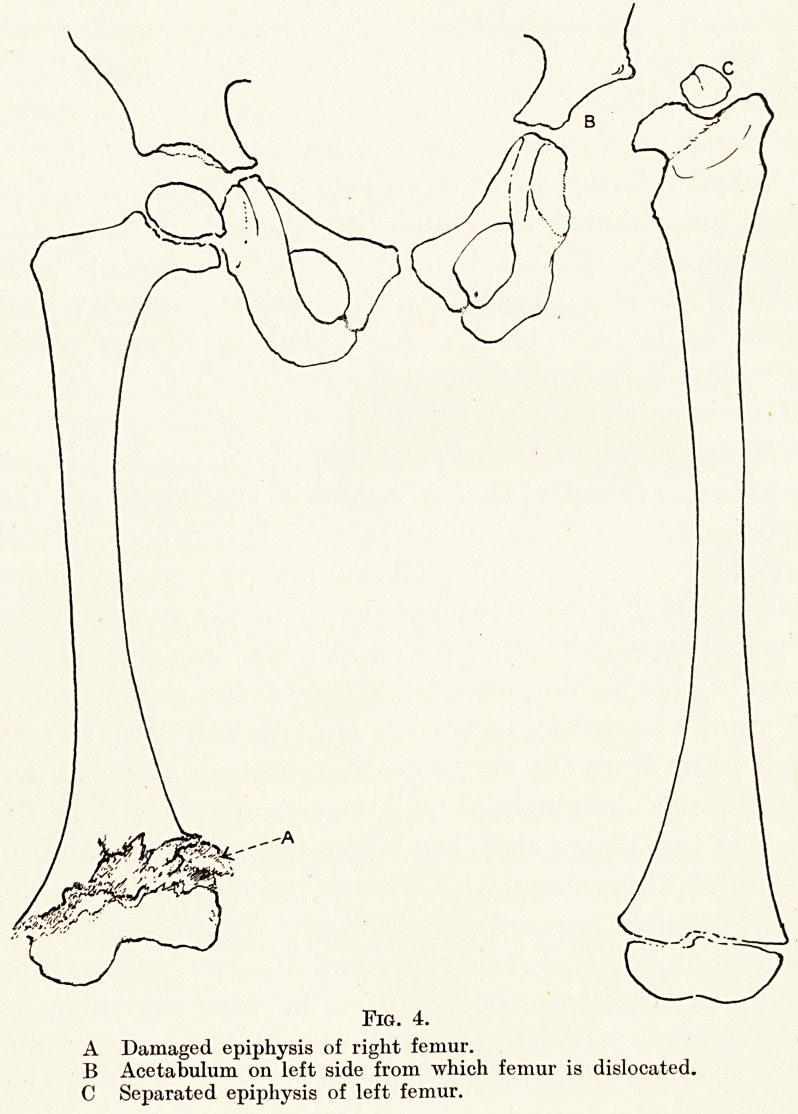# The Borderland between Surgery and Gynæcology

**Published:** 1929

**Authors:** E. W. Hey Groves

**Affiliations:** Vice-President, Royal College of Surgeons of England; Professor of Surgery, University of Bristol; Senior Surgeon, Bristol General Hospital


### THE BORDERLAND BETWEEN SURGERY AND GYNÆCOLOGY.

BY

E. W. HEY GROVES, M.S., F.R.C.S.,

*Vice-President, Royal College of Surgeons of England;*
*Professor of Surgery, University of Bristol;*
*Senior Surgeon, Bristol General Hospital.*

---

ONE of the great difficulties and dangers of the formation of any speciality in medical science is that of leaving some gap or territory between the domain cut off and the mainland from which it has been separated. Of no speciality is this more true than of gynæcology, which was the first dependency of general surgery to receive independent status.

One of the most important borderland territories between surgery and gynæcology relates to the surgery and mechanism of the female pelvis and the pain which is referred to the back, sacrum and buttocks. The operative radical treatment of flat pelvis ought to be studied by the surgeon and the accoucheur together, so as to devise, if possible, some alternative for the plan of Cæsarean section, followed by sterilization, which now holds the field.

Minor ailments which account for wrecked health and happiness in the lives of many women are the painful affections of the pelvis and its contents, the nature and treatment of which is often a matter of doubt.

For many years pain low in the back has been supposed to be due in women to uterine disorders or displacements.

Matthews Duncan invented the term "sacrache" to describe the low back pain of the sacrum in women, who often had a retroverted uterus or prolapsed ovary. But now it is generally admitted that this idea of mere uterine displacement having any serious effect is incorrect. The X-rays have demonstrated one of three lesions in nearly all the sufferers from "sacrache." These lesions are:—

1. A slipping downwards and forwards of the lumbar spine on the sacrum.

2. Some affection, sprain or arthritis of the sacro-iliac joints.

3. Some abnormal condition in the joints between the fifth lumbar vertebra and the sacrum, the common manifestation of which is an abnormal articulation of the transverse processes of the lumbar vertebra with the sacrum—the so-called sacralization of the fifth lumbar vertebra.

But it would be just as unreasonable to assume that the low back pain in women is always due to abnormalities of the lumbro-sacral spine and sacro-iliac joints as was the old assumption that it was always due to disease or displacement of the uterus and its appendages. Clearly, in these matters, either the surgeon must invade the gynæcologist's territory, or the gynæcologist the surgeon's; or—what is far more likely to advance knowledge and avoid error—the surgeon and gynæcologist must work together in territory which is their common borderland.

It is not my intention to make any further general appeal for this co-ordination, but to content myself with the relation of three recent cases of exceptional interest which illustrate very forcibly the difficulties and dangers of this borderland work.

**Case 1.**—*Severe anœmia associated with uterine fibroids, but caused by a fibroid tumour of the duodenum.*

F., aged 42, a forewoman at a factory. For some years she has suffered from irregular attacks of abdominal pain and has gradually become pale and anæmic. Menstruation has been rather irregular, lasting three to five days, loss profuse, but not painful. Has also had piles of moderate degree of severity. The abdominal pain was chiefly in the epigastrium and seemed to be associated with a moderate functional dyspepsia. In May and again in June, 1928, she had felt faint and had then passed a large loose motion as black as tar. Seen in June, she was a well-built woman, intelligent and vivacious, very pale with blanched conjuctivæ. The blood showed a cell count typical of secondary anæmia. There was a swelling in the hypogastrium the size of a fœtal head, which vaginal examination proved to be a uterine fibroid. There were a few internal hæmorrhoids. At this moment, when the patient had been examined by Dr. Taylor, Professor Rayner and myself, the obvious facts were :—

1. A profound anæmia of a secondary type pointing to a loss of blood.

2. Uterine fibroids.

3. A moderate degree of hæmorrhoids. There was also the history of her becoming faint and passing loose tarry motions on two occasions. There were three possible sources of hemorrhage, viz. the uterus, the hæmorrhoids or some intestinal lesion such as a duodenal ulcer. We therefore kept her in bed for a month, and as a preliminary measure the piles were injected with 10 per cent. carbolic acid in glycerine.

At an operation on 8th July, 1928, by Professor Rayner and myself, the abdomen was opened by a mid-line incision below the umbilicus. The whole body of the uterus was enlarged by a fibroid mass about the size of a cocoanut, and there were several smaller fibroids invading the broad ligaments. The uterus, both tubes and one ovary were removed by a supra-vaginal hysterectomy. The appendix was found to be long, thickened and adherent and contained two concretions. It was removed, and at the moment it was thought possible that the inflammation of the appendix might have been the cause of the gastric or duodenal hemorrhage.

But on enlarging the incision upwards and examining the duodenum, the latter was found to be involved by a firm elastic swelling about the size of a walnut, situated on the inferior wall of the first part of the gut. As the nature of the tumour was uncertain, and the possibility of its removal doubtful, a posterior gastro-enterostomy was done. The pylorus and first part of the duodenum, containing the tumour, were then lifted out of the wound and clamped off. A transverse incision was made across the duodenum over the middle of the tumour, through the serous and muscle coats. This allowed a firm fibroid tumour to be shelled out without injuring the mucous coat of the bowel. (Fig. 1.) The outer coats of the duodenum were sewn together by catgut. The abdomen was closed by two continuous catgut sutures for the deep and superficial sheaths of the rectus, silkworm gut for the skin and anterior rectus sheath and continuous catgut for the skin only. She made a good recovery after a rather stormy convalescence. She was very restless for forty-eight hours after the operation and burst open part of the wound, which had to be re-sutured. She left the Home four weeks after the operation, and by the early part of 1929 she was back at work, her general health being quite satisfactory. The pathologist reported that both the uterine and duodenal tumours were typical fibro-myomata of exactly similar structure.

This case illustrates the happy results that may be achieved by co-operation between the surgeon and the gynæcologist. If either of us had been working alone, the patient might have had only one lesion dealt with and a second operation at an early date would have been inevitable.

**Case 2.**—*Intestinal obstruction due to an endometrioma, growing in the wall of the pelvic colon.*

F., aged 33, unmarried. For two years she had been complaining of gradually increasing menstrual pain and marked constipation. The uterine pain was very severe on the third day of each period, and was accompanied by a sensation of bearing down in the rectum.

She was seen by Dr. Lily Baker (January, 1929), who found a mass in the rectovaginal septum and also "some curious polypoid growths" in the posterior vaginal fornix. These latter were removed and sent for section, as Dr. Lily Baker had already made a tentative diagnosis of endometrioma of the uterus invading the rectal wall.

The pathologist considered that the growths were simple

FIG. 1.

A Appearance of duodenum on exposure.
B Duodenum and pylorus in section. Tumour in situ.
C Tumour cut through centre.
D Tumour exactly as removed. Actual size.

papillomata, and showed no evidence of endometrioma or other malignant change. But her pain became more severe, and was now associated with the increasing constipation, as well as being worse than ever during the last days of each menstrual period.

At this time she came under my care, and she was subjected to careful X-ray examination after barium meals and enemas. This examination revealed nothing beyond very marked intestinal stasis, and she was sent back to her home for the time being. However, the pain, both intestinal and uterine, became steadily worse, so that the patient begged for some kind of operative relief. When undertaking this, I must admit that I regarded the case as one of abdominal neurasthenia, associated with intestinal stasis, and I undertook the laparotomy on the understanding that if no organic lesion was found, I would not do any excision or short circuiting of the colon. I very much regret that at that time I was not aware that Dr. Lily Baker had seen the case, so that I had not the advantage of her opinion as to the condition prior to the first operation.

The patient was a healthy woman, well nourished and of good colour, weighing 10 stone. The mouth was in good condition, some artificial teeth, chest normal, abdomen moved well, not distended, nothing abnormal discovered. Rectal examination revealed no stenosis, but a rather indefinite thickening opposite to and attached to the cervix uteri.

At an operation in June, 1928, through a right paramedian incision, the appendix was found to be long and narrow, but not inflamed or adherent ; the cæcum was rather dilated, as was the whole of the colon down to the pelvic portion. The sigmoid flexure of the colon, which could easily be brought up to the surface of the abdomen, showed what appeared to be a definite string carcinoma about eight inches above the pelvic floor, and one inch above the main growth was another small nodule, hard and puckered. On the pelvic floor itself (Fig. 2), in the recto-vaginal septum, was a hard mass which clearly represented an outgrowth from the cervix uteri into the rectal wall. The uterus, tubes and ovaries were otherwise normal. For the moment it seemed obvious that the patient had a growth obstructing the pelvic colon for which relief was demanded. But it was not clear whether the two growths in the colon were of the same nature as that in the recto-vaginal septum. I therefore contented myself with drawing a loop of the transverse colon out of the upper angle of the wound, the rest of which was closed. After three days the colon was opened and she made a rapid recovery, expressing herself as entirely relieved from the general abdominal pain and from the constipation. However, on further reflection, I came to the conclusion that unless something further was done, she would have to keep the colostomy permanently, and that the growth in the pelvic colon, being probably a carcinoma, would eventually prove fatal.

At the second operation on August 9th the abdomen was opened through the old incision, and fortunately was found to be free from adhesions. Both ovaries and tubes were removed

FIG. 2.

A Hard mass adherent to uterus.
B Constriction in pelvic colon.
C Nodulus secondary growth.

in order to cause involution of the endometrioma of the recto-vaginal septum. The pelvic colon was found to be exactly as in the first operation, viz. with one growth like a string carcinoma, producing great constriction and another nodule about one inch higher up. A piece of colon, six inches long, was excised, and an end-to-end anastomosis done by a double row of catgut sutures.

She made a very stormy recovery from this second operation, suffering from a succession of post-operative complications; first incessant vomiting, later severe foul-smelling diarrhœa

(through the colostomy) and then purulent bronchitis. Fortunately she recovered from all these, and in three months' time the transverse colostomy will be closed after testing the patency of the newly-restored pelvic colon. The specimen resected was very remarkable, and presented appearances which I have never seen before. Whilst from the outside it had a hard constricting growth and a small nodule one inch away, when it was cut open the mucous membrane was absolutely normal, without ulceration, inflammation or hemorrhage. On section it proved to be a typical "endometrioma," that is, an adenomyoma uteri in which islets of endometrial tissue are reproduced, surrounded by fibrous tissue which undergoes great contraction.

FIG. 3.

A Portion of pelvic colon opened after excision, showing mucous membrane intact over tumour.

B Section of the bowel cut through growth.

C Diagram to illustrate the actual relation of growth as a cause of obstruction.

It is true that the subject of endometrioma has had abundant discussion in gynæcological text-books and journals,[1, 2, 3] but it is not fully recognized in surgical literature that it is capable of actually becoming implanted upon the intestine and forming there a constricting growth which causes intestinal obstruction. Mouat has, however, recorded two cases similar to the above.

It behaves in some respects like a malignant growth. Apparently, in the first place, a portion of endometrium by its own active proliferation invades the muscular wall of the uterus, and growing there in a centrifugal direction, finally bursts through the peritoneum, where it may form a growth like a soft adenomyoma ; or else it remains buried in the rectovaginal septum. But this does not exhaust the wandering characteristics of the new growth. It has the capacity of casting off buds from its own substance into the peritoneal cavity, and these buds may become implanted upon any pelvic structure as independent new growths. This migration of endometrial buds usually occurs as the growth is breaking through the outer wall of the uterus, but it has also been suggested that it may take place through the open ends of the Fallopian tubes. In support of this latter idea is the fact that endometrial growths are commonly found in the substance of the ovaries. In the case under discussion it is easy to imagine that the loop of pelvic colon lay on the floor of the pelvis, where it would be in contact with the growth which was extending from the cervix to the rectum. While it lay there some endometrial cells became implanted in its wall in two places and then began to proliferate, and to surround themselves with fibrous tissue which steadily tied a constricting cord round the gut. An endometrioma, though having these two characteristics, viz. penetration and implantation, does not otherwise resemble a carcinoma. It does not cause metastatic growths nor does it recur when locally removed. And lastly, it has one unique characteristic related to its origin, and that is, that it withers and atrophies at the menopause or when the ovaries have been removed.

**Case 3.**—*Serious bone and joint injuries associated with difficult birth.*

My third borderland case is of quite a different kind, and deals with gross lesions suffered by an infant at birth.

FIG. 4.

A Damaged epiphysis of right femur.
B Acetabulum on left side from which femur is dislocated.
C Separated epiphysis of left femur.

X.Y., a boy aged 4 years, was born at full term by a breech presentation. The labour was a very difficult one, and the child is said to have been assisted into the world by traction over the flexed thighs. It was very severely cut and bruised especially over the outer side of the right thigh. Was at first so frail that he was not expected to survive. He walked late and badly, and when he was four years of age he was brought to the Hospital on account of lameness and unsteadiness of gait. (Fig. 4.)

He is a well-nourished child of normal intelligence. He has the pot-bellied protuberant-buttock figure of a hip dislocation. He walks with a peculiar roll and waddle of so comic a character that it seemed almost a pity to correct it. The left leg, which appears to be longer than the right, has the trochanter on a level with the anterior superior spine, and the X-ray confirms the diagnosis of a dislocation of the hip with separation of the epiphysis. The right leg is deeply scarred on its outer aspect just above the knee, and these scars are said to be the remains of the birth trauma. The leg is nearly two inches shorter than its fellow, and is greatly deformed at the knee-joint by a genu valgum. The X-ray shows that the lower epiphyseal line of the femur is very irregular and suggests that it has been seriously damaged. The damage has already resulted in arresting the growth of the right leg and distorting the shape. As the genu valgum on the already short leg caused still greater apparent shortening when the child walked, owing to a tilting upwards of the pelvis on that side, the child was extraordinarily lame.

The genu valgum could easily be corrected, and this has now been done, so that one element in his lameness is removed. At a later date, probably in about one year's time, an osteotomy will be done below the trochanter on the left side, so as to bring the line of the femur more directly under the pelvis and at the same time shortening the leg.

There are many conditions of grave surgical importance in the newly-born child which are liable to be overlooked, because they do not interfere with the general nutrition. Of these the most important are injuries of the skull and membranes of the brain, causing intracranial hemorrhage and subsequent paralysis or mental deficiency; injuries of the brachial plexus causing paralysis of the arm; and injuries of the bone and joints, as in the case just related.

The progress of medical science has been such that the development of special departments, *e.g.* that of gynæcology, is necessary and inevitable. But with the separation of such special departments there should be an organized effort to link up the neighbouring territories of work, so that the gynæcologist shall be ready to help the surgeon and the surgeon the gynæcologist, rather than leaving a borderland between two sciences in which either worker alone may have to encounter unfamiliar dangers, where his colleague might render him invaluable assistance.

My thanks are due to Dr. Lily Baker for kindly placing her notes of Case 2 at my disposal when she became aware that the patient was under my care, to Professor Rayner for his invaluable help in Case 1, and to Miss D. Pillers for the diagrams which illustrate this paper.

REFERENCES.

[1] Mouat, " Two Cases of Stricture of the Bowel by misplaced Endometrial Tissue," *British Journal of Surgery,* 1926-27, vol. xiv., p. 76.

[2] Clifford Morson, " The Pathology and Treatment of a Vesical Tumour resembling an Endometrioma," *British Journal of Surgery,* 1927-28, vol. xv., p. 264.

[3] Fraser, *Bristol Med.-Chir. Jour.,* 1927, vol. xliv., p. 113.

## Figures and Tables

**Fig. 1. f1:**
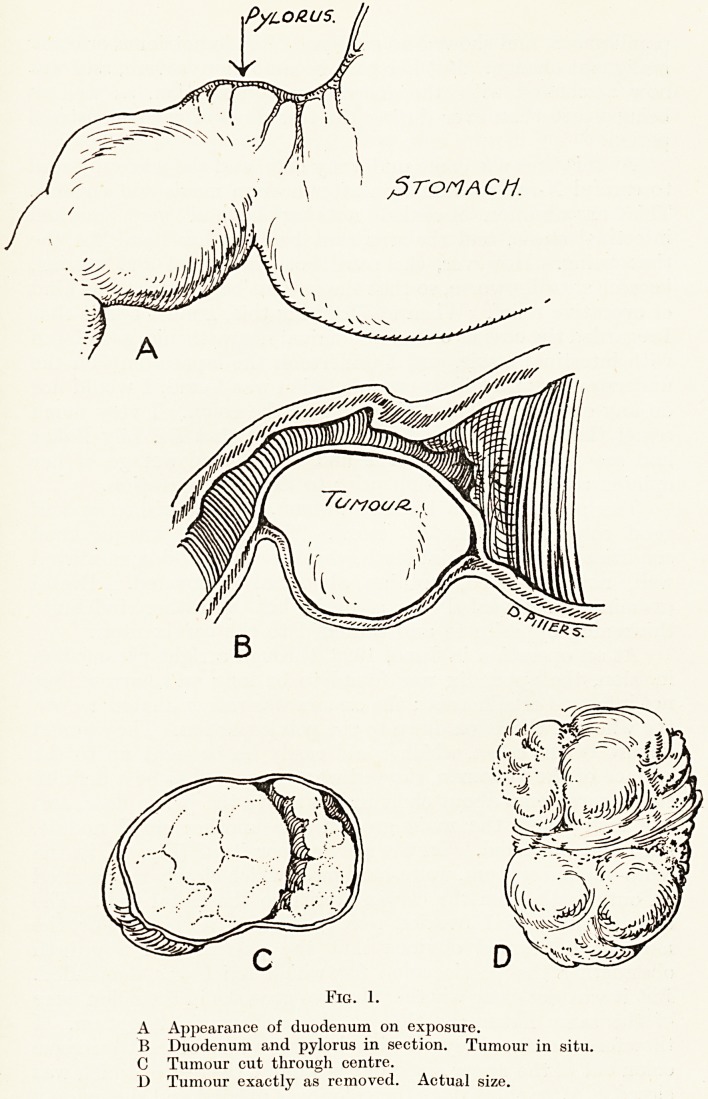


**Fig. 2. f2:**
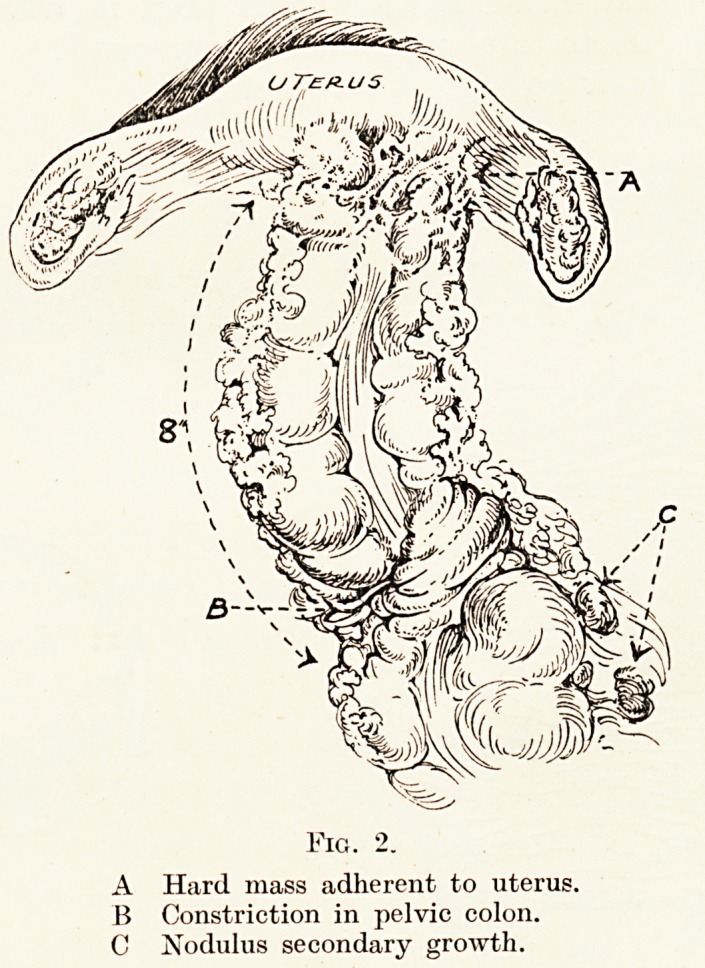


**Fig. 3. f3:**
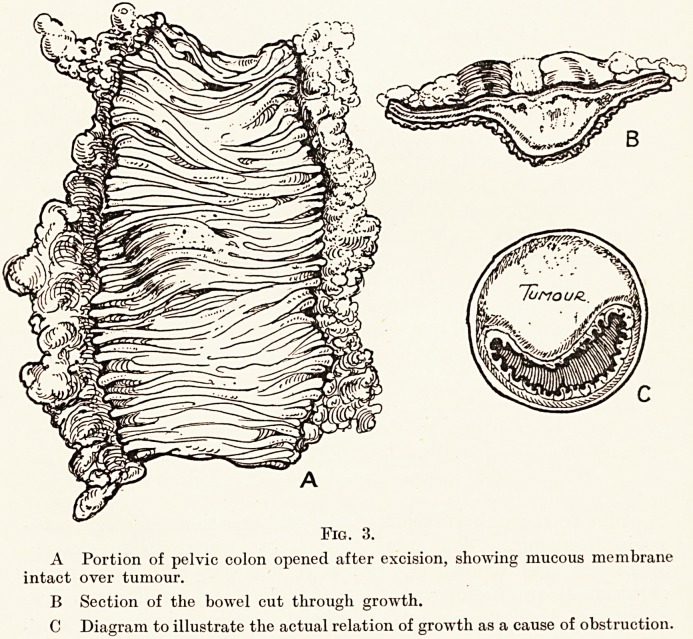


**Fig. 4. f4:**